# An Audit of Physical Health and Blood Test Monitoring in Patients Under the Care of Mersey Care NHS Foundation Trust Who Are Prescribed Lithium

**DOI:** 10.1192/bjo.2024.578

**Published:** 2024-08-01

**Authors:** Declan Hyland, Gopal Chinnari, Tawfik Elhaj-Houssen, Rose-Anne Orrell

**Affiliations:** Mersey Care NHS Foundation Trust, Liverpool, United Kingdom

## Abstract

**Aims:**

Lithium is clinically indicated for use in the UK for treatment and prophylaxis of mania, bipolar disorder, recurrent depressive disorder and aggressive of self-harming behaviour. In patients who are prescribed lithium, several physical health checks and blood tests must be completed on a regular basis to ensure lithium remains safe and appropriate to continue. Lithium has a narrow therapeutic index and so close monitoring of serum lithium level is required.

This audit aimed to establish whether Mersey Care NHS Foundation Trust’s physical health check and blood test monitoring of patients prescribed lithium is in keeping with NICE guidelines and determine how the Trust’s performance compared with national performance as identified by the Prescribing Observatory for Mental Health (POMH) lithium audit.

**Methods:**

A total of 127 patients under the care of the Trust who were prescribed lithium were identified. The POMH lithium audit tool was used to capture data for each patient as Mersey Care NHS Foundation Trust was participating in the POMH lithium audit. Each patient's electronic record was scrutinised to determine whether the following were measured every six months during maintenance treatment – Thyroid Function Tests (TFTs), serum calcium level, estimated Glomerular Filtration Rate (eGFR) and serum lithium level, and whether the patient had a weight/body mass index (BMI)/waist circumference within the last 12 months.

**Results:**

Of the 127 lithium patients included in the audit, 64% had a serum calcium level done every six months, 78% had TFTs done every six months, 83% had an eGFR done every six months, and 87% had a serum lithium level done every six months. 71% of patients had a weight/BMI/waist circumference within the last 12 months.

**Conclusion:**

Trust performance for TFT monitoring and weight/BMI/waist circumference was above the national compliance level reported in the POMH lithium audit; Trust performance for serum lithium level, eGFR and serum calcium level was below the national compliance level. There is a need to ensure that medical and nursing staff are aware of the physical health checks and blood test monitoring required for patients maintained on lithium. A Quality Performance Alert will be sent to medical and nursing staff in the Trust to raise awareness and lithium monitoring will be included in the junior doctor Trust induction. Future auditing of Trust performance on physical health check and blood test monitoring for patients maintained on lithium will be conducted.

